# Daytime Solar Heating Controls Downy Mildew *Peronospora belbahrii* in Sweet Basil

**DOI:** 10.1371/journal.pone.0126103

**Published:** 2015-05-20

**Authors:** Yigal Cohen, Avia E. Rubin

**Affiliations:** The Mina & Everard Goodman Faculty of Life Sciences, Bar-Ilan University, Ramat Gan 5290002, Israel; Agriculture and Agri-Food Canada, CANADA

## Abstract

The biotrophic oomycete *Peronospora belbahrii* causes a devastating downy mildew disease in sweet basil. Due to the lack of resistant cultivars current control measures rely heavily on fungicides. However, resistance to fungicides and strict regulation on their deployment greatly restrict their use. Here we report on a ‘green’ method to control this disease. Growth chamber studies showed that *P*. *belbahrii* could hardly withstand exposure to high temperatures; exposure of spores, infected leaves, or infected plants to 35-45°C for 6-9 hours suppressed its survival. Therefore, daytime solar heating was employed in the field to control the downy mildew disease it causes in basil. Covering growth houses of sweet basil already infected with downy mildew with transparent infra-red-impermeable, transparent polyethylene sheets raised the daily maximal temperature during sunny hours by 11-22°C reaching 40-58°C (greenhouse effect). Such coverage, applied for a few hours during 1-3 consecutive days, had a detrimental effect on the survival of *P*. *belbahrii*: killing the pathogen and/or suppressing disease progress while enhancing growth of the host basil plants.

## Introduction

*Peronospora belbahrii* Thines has recently become a major disease of sweet basil (*Ocimum basilicum* L) in many countries [1, 2]. In Israel, it was first observed in November 2011. The origin of the pathogen was not discovered. By the summer of 2012, the disease had appeared throughout the country, causing major economic damage [3]. Within one year of use, the fungicide mefenoxam became ineffective in controlling the disease due to the appearance of resistant isolates [3].

Current control measures rely mainly on applying fungicides. In Israel, field experiments showed that the best performing fungicides against mefenoxam-sensitive isolates are (in order) mefenoxam, mandipropamid, dimethomorph, and azoxystrobin; whereas against mefenoxam-resistant isolates the most effective fungicides are mandipropamid, dimethomorph, signum, pyraclostrobin and azoxystrobin (Y. Cohen, Y. Ben-Naim, and M. Vaknin, unpublished data). Because of the strict regulations imposed by the authorities on applications of fungicides to basil, we looked for alternative methods to combat this disease. One effective method was illumination during the night which inhibited sporulation of the pathogen [4].

Basil crops in Israel are frequently grown under cover, a fact that greatly enhances the feasibility of also using daytime solar heating as a control measure.

The objectives of the present study were to investigate the effects of heat on survival of *P*. *belbahrii* in growth chambers and then to implement the results for disease control under commercial production conditions in the field.

## Materials and Methods

### Plants

The sweet basil cultivar Peri (Volcani Center for Agricultural Research, Newe Ya’ar, Israel) was used in all experiments. Plants were grown in 0.5L pots filled with peat: vermiculite (1:1, v/v) in the greenhouse (night/day temperature 18°C/32°C). Plants were used for experiments at the 8–14 leaf stage, unless stated otherwise.

### Pathogen and inoculation

A mefenoxam-resisatnt isolate of *P*. *belbahrii* was used in all experiments. This isolate was collected in Northern Israel in late 2012. It was maintained by repeated inoculations of whole plants in a growth chamber at 25°C. Plants were inoculated by spraying a conidial suspension (5000 conidia/ml) on their upper leaf surfaces until run off. Inoculated plants were kept in a dew chamber (18°C) in the dark for 20h to promote infection, and then transferred to a growth chamber at 25°C with continuous illumination (cool white and warm white fluorescent lamps, 60 μmole m^2^ s^-1^) to allow for fungal colonization but not sporulation. At 6dpi (days post inoculation) plants, exhibiting chlorotic lesions on their leaves, were returned to the dew chamber for 20h to induce sporulation of the pathogen. Heavy sporulation occurred on the lower leaf surfaces, more on upper (younger) than on the lower (older) leaves.

### Heat tolerance of mycelium in intact plants

Survival of the pathogen in infected leaf tissue was studied as follows: infected basil plants (12 to 14 leaf stage, 6 plants per treatment) were transferred at 2–9 dpi for 3, 6, 9, 17 or 27 h into a series of growth chambers calibrated to 20, 25, 30, 35, 40, or 45°C (all ±0.5°C, RH 40–80%) with continuous CW fluorescent illumination (~30 μmole m^-2^ s^-1^). At the end of the heat treatment, plants were returned to 25°C (as above) for at least one day, or until 7dpi, and thereafter allowed to sporulate overnight in a dew chamber (18°C) in the dark. Survivability of the pathogen was determined by counting the number of sporulating leaves out of the total number of leaves per plant. The results were expressed as % sporulating leaves (see below).

To test the sensitivity of mycelium to RH (relative humidity), the top 6 leaves were detached from infected plants (12 to 14 leaf stage) at 5dpi and exposed for 24h, in special containers, to various RH levels ranging from 22% to 100% at 20–30°C. Leaves were then removed from the containers, placed in moistened Petri dishes (14cm) at 25°C under continuous illumination for 24h, and thereafter allowed to sporulate for 20h at 18°C in darkness. The intensity of sporulation serves as a criterion for mycelium survival.

### Heat tolerance of mycelium in cut branches

Basil branches (20–25 leaves) infected with downy mildew were collected from the field, washed thoroughly with water to remove the existing spores, placed with their stem base in 2L plastic cylinders (30 cm height) containing 1L water (10 branched per cylinder per treatment) and exposed to 25–45°C for 3 h or to 15–40°C for 6, 13, 20 or 32h. After exposure, the water was replaced with fresh tap water; the branches were returned to 25°C for one day and thereafter placed overnight in a dew chamber at 18°C in the dark to allow for sporulation of the pathogen. The proportion of sporulating leaves in each branch was visually determined and served to assess the survivability of the mycelium inside the leaves.

### Heat tolerance of attached spores in moisture-saturated air

Infected basil plants at 6 dpi were placed overnight in a dew chamber (18°C) in the dark to induce sporulation of the pathogen on the infected leaves. The top four leaves carrying abundant spores were detached and placed, lower surface upward, on wet filter paper inside 14cm Petri dishes. Dishes were sealed and exposed to heat treatments, 3 dishes per treatment. In other experiments, whole sporulating plants were sealed in transparent plastic boxes and exposed to heat treatments (4 plants per treatment). At 6, 12 and 24 h, twelve sporulating leaves and at 22 and 72 h four whole plants were removed from dishes or boxes, respectively. The spores were collected into ice-cold water (~4°C), calibrated to 5000 spores/ml and sprayed onto 4–6 basil plants (12 to 14 leaf stage). Inoculated plants were placed overnight in a dew chamber (as above) to allow for infection, and thereafter placed in a growth chamber at 25°C under continuous light to allow for disease development. At 6 dpi, plants were placed in a dew chamber (as above) to allow for fungal sporulation. Sporulation of the pathogen was visually determined by counting the number of sporulating leaves out of the total number of leaves per plant.

.

### Heat tolerance of attached spores at ambient air

Infected plants at 6 dpi were placed overnight in a dew chamber (18°C) in the dark to induce sporulation of the pathogen. The sporulating plants were exposed as such (4 plants per treatment) to 20, 25, 30, 35, 40 or 45°C for 3, 22, 48, 72 or 96 h in a series of growth chambers as described above. After exposure, the spores which remained on the leaf surfaces were collected into ice-cold water, calibrated to 5000 spores/ml and sprayed onto 4–6 basil plants (12 to 14 leaf stage). The inoculated plants were placed in a dew chamber to allow infection and then at 25°C under light to allow for disease development as described above. Heat tolerance of the attached spores was determined by counting the number of sporulating leaves out of the total number of leaves per plant inoculated.

### Heat tolerance of wind-dispersed spores

Healthy basil plants (12 to 14 leaf stage, 50–80 plants per experiment) were exposed to spore shower for 4h (9 am to 1 pm) by placing them in a net house in which adults basil plants, heavily infected with downy mildew, were growing. The trap plants, carrying spores of *P*.*belbahrii* on their leaf surfaces, were brought to the laboratory, exposed to 15, 20, and 28°C for 0, 24 and 96 h (first experiment), to 25 and 30°C for 0, 24, 48, 84 and 96 h (second experiment), to 15, 20, 25, 30, 35 and 40°C for 31 h (third experiment) and 72 h (fourth experiment) in growth chambers as described above. Six plants per treatment were used in all four experiments. Afterward the plants were placed in a dew chamber to allow for infection and then at 25°C under light to allow for disease development. At 6 dpi, plants were returned to the dew chamber (as above) to allow for fungal sporulation. The sporulation of the pathogen, which was visually determined by counting the number of sporulating leaves out of the total number of leaves per plant, served as a criterion for survival of the wind-dispersed spores.

### Heat tolerance of splash-dispersed spores

Healthy basil plants (12 to 14 leaf stage) were spray-inoculated onto their upper leaf surfaces with a fresh spore suspension (50,000 spores/ml). Inoculated plants were immediately placed in a strongly ventilated hood to dry off the inoculum spray droplets (20–30 minutes). The inoculated plants were then exposed to 20, 25, 30, 35, 40 and 45°C for 3, 6, 9, 20 and 55 hours (4 plants per treatment) in a series of growth chambers in ambient humidity. Afterward, plants were transferred into a dew chamber (as above) to allow for spore infection and then incubated at 25°C to allow fungal sporulation. The sporulation of the pathogen was visually determined by counting the number of sporulating leaves out of the total number of leaves per plant inoculated. It served as a criterion for survival of the splash-dispersed spores. In other experiments, survival of splashed-dispersed spores *on planta* and *in vitro* was compared. For this purpose healthy basil plants and the bottom of opened 14-cm diameter Petri dishes were sprayed with fresh spore suspension (50,000 spores/ml) and allowed immediately to dry off as above. Inoculated plants and Petri dishes (4 plants and 4 dishes per treatment) were then exposed to 20, 25, 30, 35, 40 and 45°C for 9h. Afterward, spores were collected from each Petri dish into ice-cold water and sprayed-inoculated onto a single basil plant. All plants were then transferred to a dew chamber to allow for spore infection, then incubated at 25°C to allow for disease development, and at 6 dpi, placed in a dew chamber to enable fungal sporulation.

### Heat tolerance of attached spores at various RHs

Infected basil plants at 6 dpi were placed overnight in a dew chamber (18°C) in the dark to induce sporulation of the pathogen on the infected leaves. The top four leaves carrying abundant spores were detached and exposed to various RHs. The various RH levels were produced in sealed transparent containers (500 ml) each containing a different saturated salt solution which produces a certain RH in the container, from 11% to 100% [5]. A special device was constructed on which the leaves were hanged to avoid them contacting the salt solution. The containers, with the experimental leaves, were incubated at 20, 30, or 40°C for 24h or 48h and thereafter the spores were collected and tested for their infectivity on 4 plants per treatment as described above.

### Daytime solar heating experiments

The effects of daytime solar heating on survival of *P*. *belbahrii* and development of downy mildew were tested in 8 experiments A–H conducted during 2013–2014 at Bar-Ilan University Farm (32° 4' 9" N / 34° 50' 35" E). Basil plants were grown in two types of net-houses, large (6×45m) or small (6×6m). Solar heating was achieved by covering a net house with a transparent, IR (infra-red impermeable) polyethylene (PE) sheet (Arava type, 100μ width, supplied by Ginegar Plastic Products, Israel or by Polytiv Ltd, Israel). In the first series of experiments the infected plants grew in containers (1.2×0.6×0.2m) filled with a soil mixture, whereas in the second series, infected basil plants grew in soil.

#### Experiment A

On October 10, 2013, about 800 ten-leaf basil plants were planted in each of two large net-houses NH3 and NH6. The houses were covered with PE after planting but the west and east sides were left free of PE to allow ventilation. Plants were artificially inoculated with *P*. *belbahrii* at two weeks after planting. By mid-November heavy epidemic of downy mildew had developed in both houses. On November 19, the east and west sides of NH3 were sealed with PE to allow the temperature to rise, whereas the east and west sides of NH6 remained open to moderate the rise in temperature. Temperature and relative humidity were monitored within the canopy with the aid of HOBO data logger UX100-003 (Onset Computer Corp. USA). One hundred infected leaves showing sporulation of *P*. *belbahrii* were sampled at 8 am from each house at 2, 4 and 6 days after PE enclosure. At each sampling date, the spores were collected from all leaves into ice-cold water, calibrated to 5,000 spores/ml, and sprayed onto 8 basil plants (12 to 14 leaf stage) to examine their infectivity as described above. The sampled leaves were then washed with excessive water to remove the remaining spores, blotted dry and placed on wet filter paper in 20×20×2 cm plastic dishes at 20°C (12h light/day) for 24h to allow re-sporulation of the pathogen. The capacity to re-sporulate was visually estimated in each leaf using the visual 0–3 scale described before [4] and mean sporulation intensity was calculated for all sampled leaves.

#### Experiment B

In mid-February 2014 about 800 six-leaf plants were planted in NH6. The house was covered with PE but the west and east sides were left open. The high humidity and moderate rise in temperature facilitates a natural epidemic of downy mildew. By March 18 plants had 10–12 leaves and were all fully infected. To examine the effect of solar heating on survival of *P*.*belbahrii*, both sides of the house were enclosed with PE on March 18 and remained sealed for 12 days, until March 30. Leaves were first sampled just before enclosure and thereafter, starting at 3 days after enclosure, every day (except Saturday) for 9 days. Temperature and relative humidity were monitored within the canopy with the aid of HOBO data logger. Similar monitoring was done in adjacent NH3, which was left open on both sides. On March 30, and again on March 31, forty healthy potted basil plants (10 to 12 leaf stage) were evenly distributed between the rows inside NH6 and kept there from 8am until 1pm to trap the air-borne spores dispersed from the infected plants. The trap plants were thereafter placed in a dew chamber to allow infection, incubated at 25°C under continuous light for 6 days and then allowed to sporulate in a dew chamber at 18°C in the dark for 20h.

#### Experiment C

On April 9, 2014, about 800 six-leaf plants were planted in PE-free NH6. Plants were inoculated 13 day after planting (22.4.2014) with a mefenoxam-resistant isolate of *P*. *belbahrii*. At 17dpi (9.5.2014) disease records were taken and the house was fully covered with PE for 3 days during 11.5.2014–14.5.2014 (19-21dpi). Disease records were taken again at 33 dpi (28.5.2014), two weeks after PE had removed. Temperature and relative humidity were monitored within the canopy with the aid of HOBO data logger. One hour before enclosure, and at 1, 2, and 3 days after enclosure, 50 leaves were collected from the infected plants, brought to the laboratory, spores were collected into ice-cold water, calibrated to 5000 spores/ml and inoculated onto 8 healthy, potted, 12-14-leaf plants, to test their viability/infectivity. The sampled leaves were then washed with excessive water to remove the remaining spores, blotted dry and placed for 24h on a moist filter paper in petri dishes at 20°C (12h light/day) to allow for re-sporulation, which served as a criterion for mycelium viability. Disease development at 33 dpi (28.5.2014) served to evaluate the long-term effect of daytime solar heating on disease progress and plant development.

#### Experiment D

Six-leaf plants were inoculated with *P*. *belbahrii* on 8.5.2014 and incubated at 25°C under continuous light. At 3 dpi (11.5.2014) plants were transplanted in soil in four small (6×6 m) net-houses, 50 plants per house. House A served as PE-uncovered control while the other three houses were covered with PE. The PE was removed from Houses C, D and E at 4, 9 and 11 days after planting, respectively. Temperature and relative humidity were monitored at the plant level with the aid of HOBO data logger. At 14 days after planting (17 dpi) plants were uprooted, the number of sporulating leaves per plant was counted, the root was cut away and the shoot fresh weight was taken.

#### Experiment E

Ten-leaf plants were inoculated on 7.6.2014 and incubated at 25°C under continuous light. At 3 dpi (10.6.2014) plants were transplanted in soil in the 4 net-houses, 40 plants per house. Houses C, D and E were covered with PE immediately after planting while House B served as PE-uncovered control. The PE was removed from Houses C, D and E at 1, 2 and 3, days after planting, respectively. Temperature and relative humidity were monitored at the plant level with the aid of HOBO data logger. At 5 days after planting (8 dpi) plants were uprooted, placed in a dew chamber overnight to facilitate sporulation and the number of sporulating leaves per plant was counted at 9 dpi.

#### Experiment F

Ten-leaf plants were inoculated on 22.6.2014 and incubated at 25°C under continuous light. At 6 dpi (29.6.2014) they were transplanted in soil in four small net-houses, about 140 plants per house. On July 1^st^, 2^nd^ and 3^rd^, Houses B, C, and D were covered with PE for 2, 4, or 6 hours, respectively, starting at 8am. House A served as PE-uncovered control. Temperature and relative humidity were monitored at the plant level with the aid of HOBO data logger. Eighty inoculated plants were left at 25°C and served as indoor controls. At 23 dpi, plants were uprooted, the shoot fresh weight (5 shoots per replicate) was taken. Shoots were placed in a dew chamber overnight to facilitate sporulation and the number of sporulating leaves per plant was counted at 24 dpi.

#### Experiment G

One hundred and fifty 10-leaf potted plants were inoculated with *P*. *belbahrii* and incubated at 25°C under continuous light. At 6 dpi (12.8.2014, 8am) 40 plants were transferred at 8 am into a plastic-house F covered with PE while other plants remained at 25°C as controls. Ten plants were taken out at 10 am, 11 am and 12 am and placed at 25°C. At 4pm, the exposed and the control plants were placed in a dew-chamber for 20h to allow fungal sporulation. Survival of *P*. *belbahrii* was determined by counting the number of sporulating leaves per plant. The experiment was repeated at 7 dpi (13.8.2014).

To examine the effect of solar heating on spore survival, 50 infected plants were placed at 7 dpi in a dew chamber to induce sporulation. The sporulating plants were placed in plastic house F on 14.8.2014 at 10:30 am and removed after exposure of 30, 60 or 90 minutes. The spores were collected and used to inoculate healthy basil plants. Sporulation on the inoculated plants a week later served to determine the survival of the attached spores after solar heating.

#### Experiment H

Five hundred 6-leaf plants were planted on 20.9.2014 in soil in Houses A-E,100 plants/house.On 29.9.2014, when reached the 10-leaf stage, the plants were inoculated with *P*.*belbahrii*. At 6 dpi (5.10.2014), when plants became uniformly infected (mild chlorotic lesions) with downy mildew, houses B, C, D and E were covered with PE for 2, 4, 6, or 7 hours, respectively, starting at 8:30 am. House A served as PE-uncovered control. Temperature and relative humidity were monitored at the plant level with the aid of HOBO data logger. At 7dpi, 40 healthy potted basil plants at the10-leaf stage were put in each house for 24h to serve as traps for surviving spores of *P*.*belharii*. The trap plants were placed in a dew chamber for 20h and then incubated at 25°C (see above) and the sporulation of the pathogen was recorded 7 days later. Percentage of sporulating leaves in Houses A-E was recorded two days after exposure of plants to heat (8 dpi).

### Data analysis

Laboratory experiments were performed two or more times with 4–6 plants (10 to 12 leaf stage) per treatment (unless stated otherwise). Results from repeated experiments showed little variation. Data from one experiment in which the variations among replicates within treatments were minimal are given. Eight experiments were conducted in the field. Analysis of variance of laboratory and field experiments was done using JMP software (SAS Institute). Statistics are given as means and standard deviations of the means. Significant difference between means were calculated for α = 0.05 [Tukey’s HSD (honest significant difference) test].

## Results

### Laboratory experiments

#### Heat tolerance of mycelium in intact plants and detached leaves

**Fig 1:**
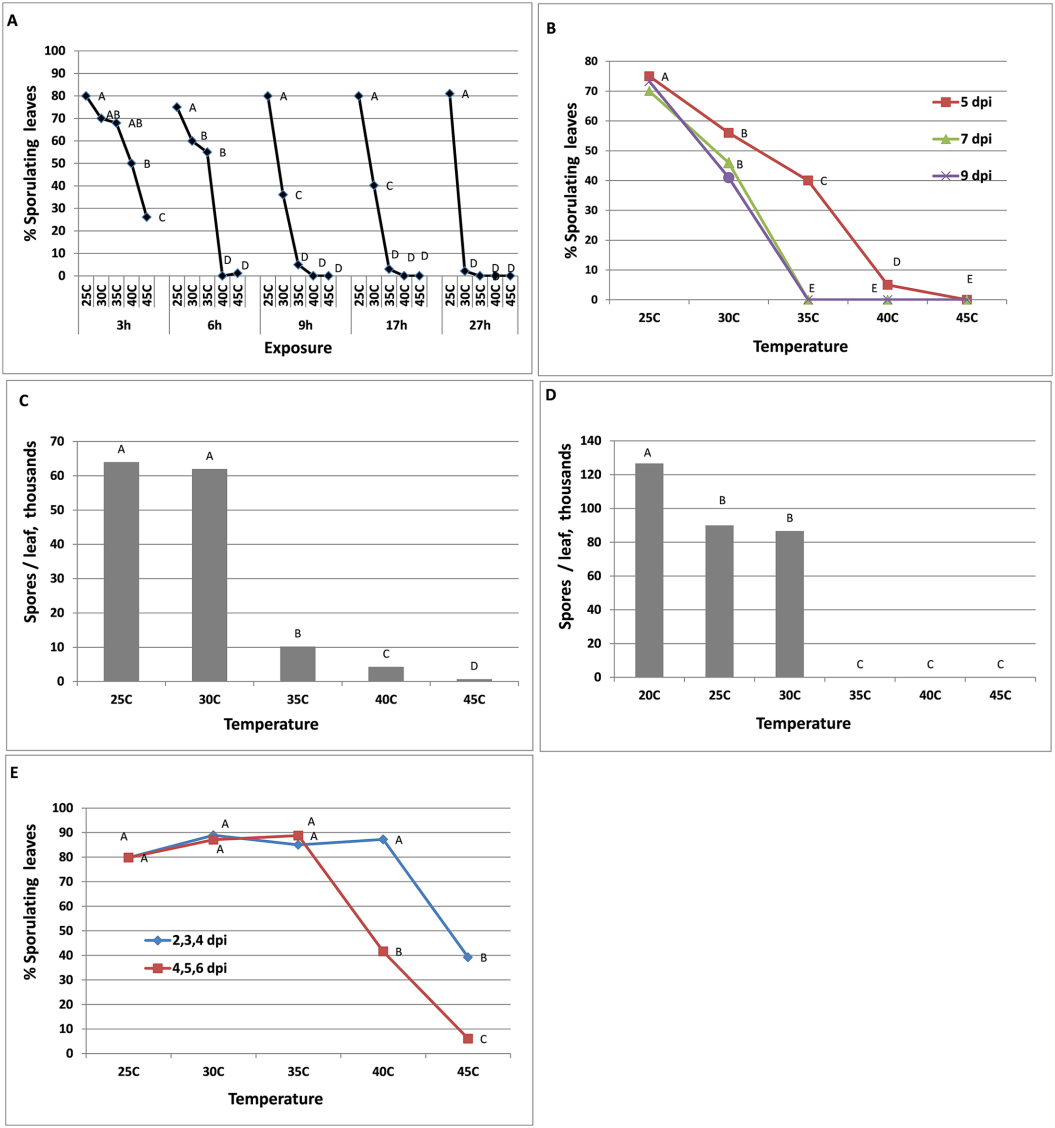
The suppressive effect of heat treatments at 25, 30, 35, 40 and 45°C on sporulation capacity of mycelium of *Peronospora belbahrii* in inoculated basil leaves. **A-** Capacity of *P*. *belbahrii* to sporulate on infected basil plants after exposure to heat treatments for 3, 6, 9, 17 and 27 h at 7 dpi. **B-** The effect of mycelium age on the capacity of *P*. *belbahrii* to sporulate on infected basil plants after exposure to heat treatments for 9 h at 5, 7, 9 dpi. **C-** Spore production in leaves of intact basil plants exposed to heat treatments for 12 h at 5 dpi. **D-**Re-sporulation in infected leaves collected from the field, spores being first washed out and exposed to heat treatments for 20 h. **E-** Capacitiy of *P*. *belbahrii* to sporulate on infected basil plants after 3 consecutive daily exposures of 3 hours each to heat treatments at 7 dpi. Exposures were applied at 2, 3 and 4 dpi or 4, 5 and 6 dpi. Different letters indicate significant differences between means at α = 0.05 [Tukey HSD (honest significant difference) test].

Infected plants at 7 dpi, were exposed to heat for 3–27 hours and then placed in a dew chamber to allow for sporulation. Results shown in Fig 1A indicate that exposure to heat had a significant negative effect on mycelium survivability (capacity to sporulate) of *P*. *belbahrii* in intact infected leaves. The longer was the exposure period and higher was the temperature the greater was the suppression of sporulation (Fig 1A). Six or more hours of exposure to 40–45°C were detrimental; 17h at 35–45°C; 27h at 30–45°C (Fig 1A). Prolong incubation in moist condition under illumination did not restore sporulation on the exposed plants.

The effect of heat on survival of the mycelium in intact plants was dependent on the interval period lapsed from inoculation to exposure (colonization period). Plants at 5, 7 or 9 dpi were exposed to heat of 9 hours and then placed in a dew chamber to induce sporulation. Results presented in Fig 1B show that the mycelium at 5 dpi was significantly less sensitive to 35°C or 40°C compared to mycelium at 7 or 9 dpi. However, mycelia at all three ages were killed at 45°C (Fig 1B).

Infected plants were exposed at 5 dpi to 12h of heat and allowed to sporulate at 7 dpi. Spore yield per leaf was not affected by exposure to 30°C but had gradually and significantly declined after exposure to 35–45°C (Fig 1C). Infected leaves were collected from the field, washed with water to remove existing spores and exposed to 35–45°C for 20h, then placed in conditions favorable for sporulation. Resporulation occurred after exposure to 25–30°C but not after exposure to 35–45°C (Fig 1D). Three consecutive daily exposures to heat, 3h each, were significantly more suppressive to the pathogen when applied to intact plants at 4, 5 and 6 dpi than if applied at 2, 3 and 4 dpi (Fig 1E).

Infected leaves exposed at 5 dpi to 22–100% RH for 24h at 20, 25 or 30°C lost turgor at low RH s but all had recovered and showed abundant sporulation when transferred to high humidity, suggesting that RH has no effect on survival of the mycelium inside the infected leaves.

#### Heat tolerance of mycelium in cut branches

**Fig 2:**
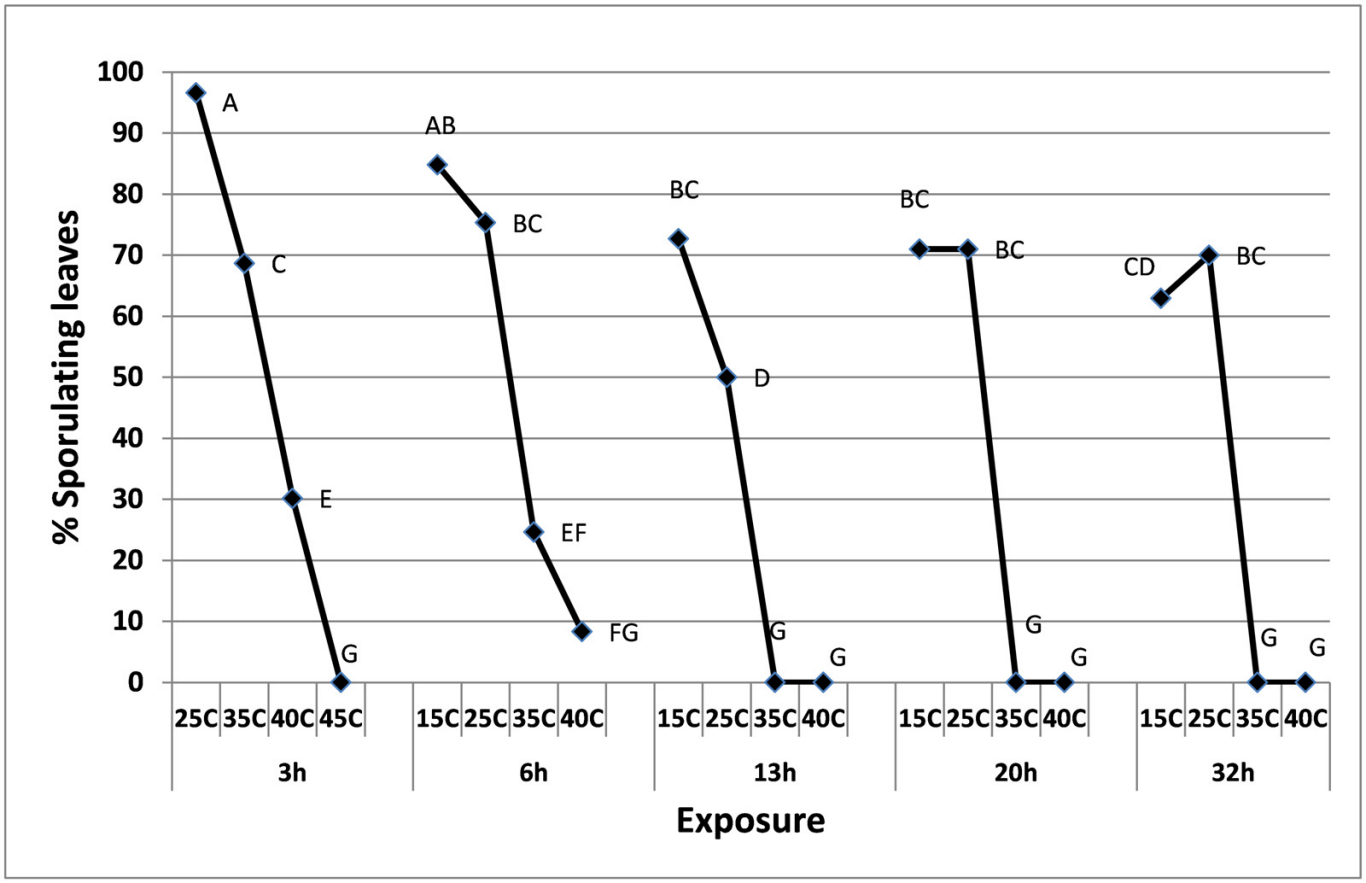
The suppressive effects of heat treatments on sporulation capacity of mycelium of *P*. *belbahrii* in leaves of cut basil branches. Different letters on graphs indicate significant difference between means at α = 0.05 (Tukey’s HSD test).

Survival of *P*. *belbahrii* in infected cut basil branches is shown Fig 2. Overall, the higher was the temperature and longer was the exposure period, the greater was the suppression of sporulation. Three hours of heat were detrimental to the pathogen at 45°C and 13h at 35–40°C. Branches exhibited no visible stress after heat treatment.

#### Heat tolerance of attached spores in moisture-saturated air

**Fig 3:**
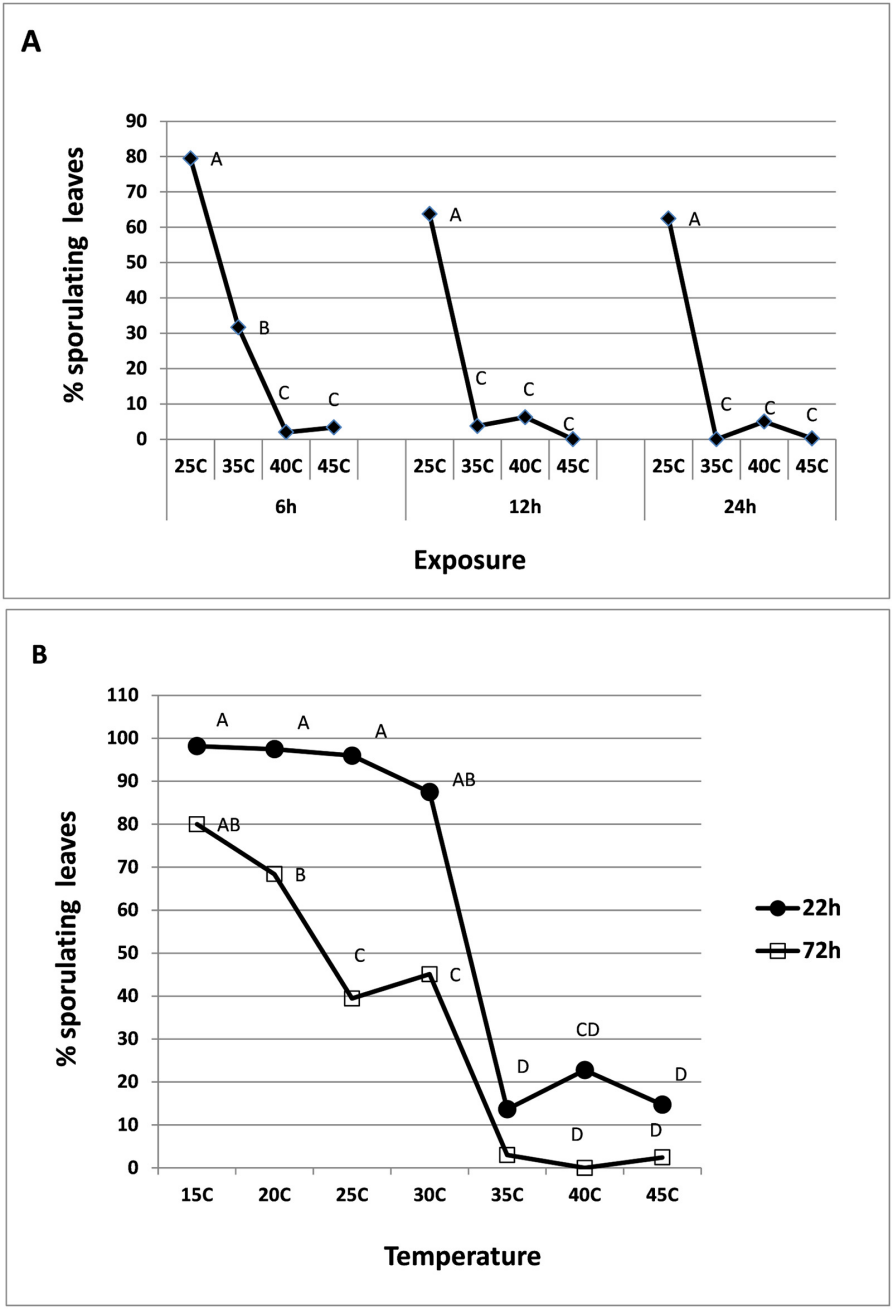
Survival of *P*. *belbharii* spores inoculated on basil plants, 7 dpi. Spores derived from **A-** detached inoculated leaves (7 dpi) kept in sealed Petri dishes and exposed to 25, 35, 40 and 45°C, respectively for 6, 12, or 24h each and **B-** whole inoculated plants (7 dpi) kept in sealed transparent plastic boxes exposed to 15, 20, 25, 30, 35, 40 and 45°C, respectively for 22 or 72 h each. Different Letters indicate significant differences between means at α = 0.05 (Tukey’s HSD test).

Results presented in Fig 3 show that heat significantly reduced survival of attached spores. Survival declined as temperature and exposure time increased. Exposure to 35°C in Petri dishes for 6, 12 or 24h reduced spore viability by 60, 94, and 100%, respectively (Fig 3A). In moistened plastic boxes, spores exposed to 35–45°C for 22h retained 14–23% viability and only 0–3% after exposure of 72h (Fig 3B).

#### Heat tolerance of attached spores in ambient air humidity

**Fig 4:**
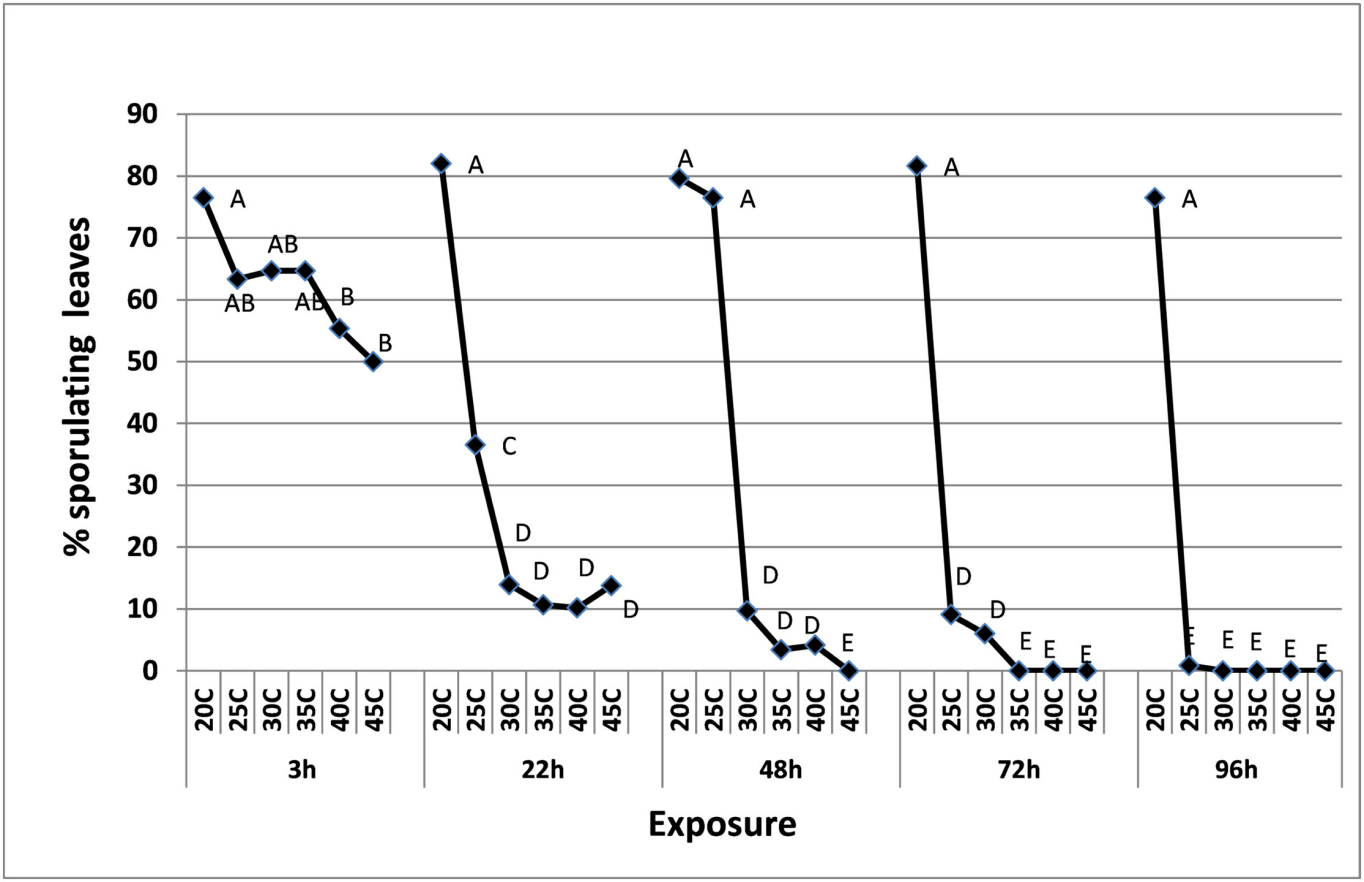
Survival of *P*. *belbharii* spores inoculated on basil plants, 7 dpi. Spores derived from whole inoculated plants (7 dpi) exposed to 20, 25, 30, 35, 40 and 45°C, respectively for 3, 22, 48, 72 and 96 h each. Different Letters indicate significant differences between means at α = 0.05 (Tukey’s HSD test).

Infected potted plants carrying abundant fresh spores were exposed to a series of temperatures (20–45°C) for 3-96h. Thereafter, the spores were collected and inoculated onto healthy basil plants to test their survivability. As shown in Fig 4, spore survival was strongly affected by temperature and duration of exposure. The longer was the exposure period and higher was the temperature the weaker was the infectious capacity of the spores. Spores collected after 3h survived well at all temperatures, although viability declined at 40 and 45°C. A sharp decline in spore survival occurred at 22h and more so at 48-96h. No survival was seen after exposure to 45°C for 48h; 35–45°C for 72h; 25–45°C for 96h (Fig 4).

#### Heat tolerance of wind-dispersed spores

**Fig 5:**
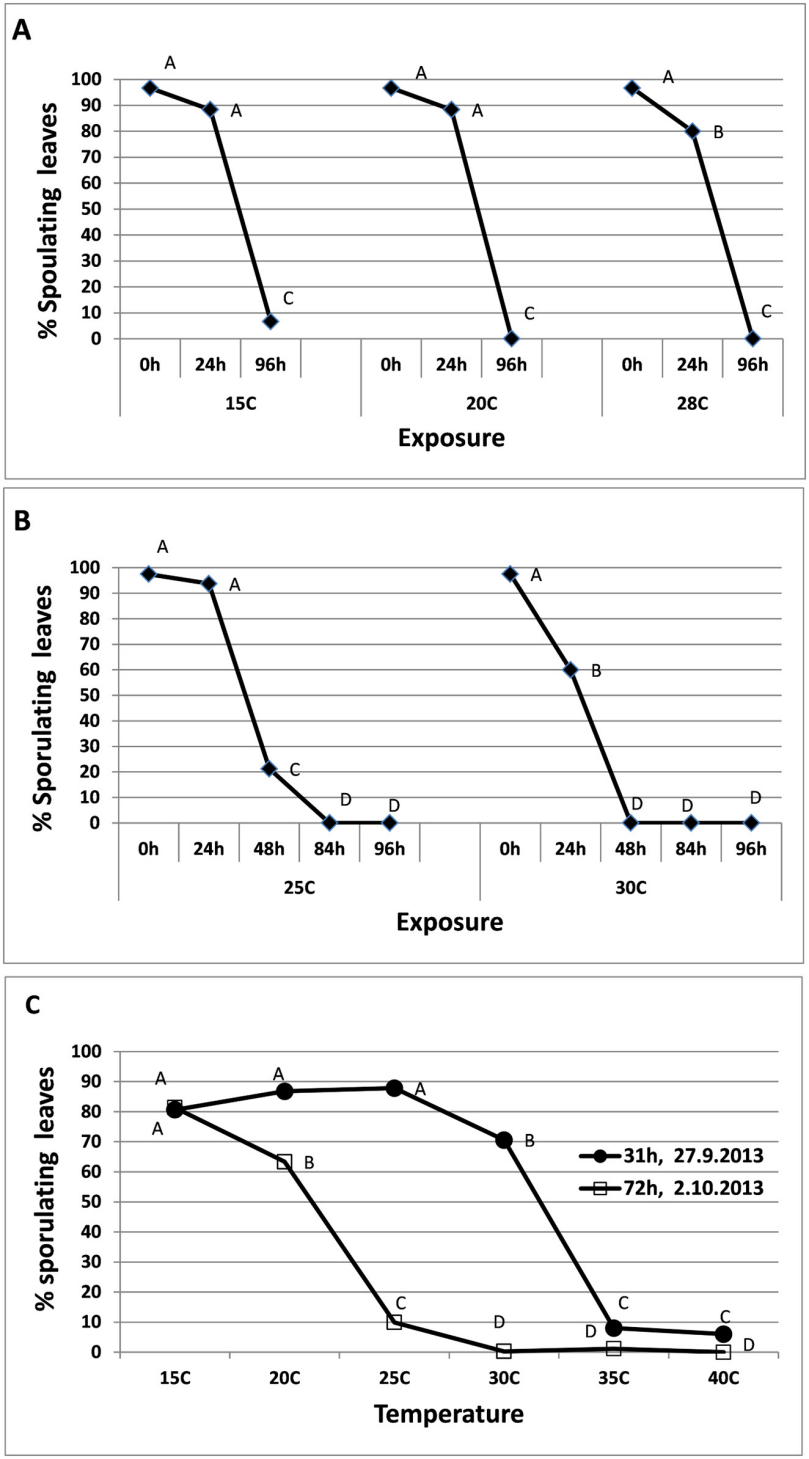
A-C- Survival of wind-dispersed spores of *P*. *belbahrii* on leaf surfaces of healthy basil plants exposed to various temperatures for various periods of time. Different letters on graphs in each chart indicate significant difference between means at α = 0.05 (Tukey’s HSD test).

Four experiments were conducted in order to determine the ability of naturally-dispersed spores to survive on plant leaf surface. In all, healthy plants were exposed to spore showers from other affected plants for 4 hours, returned to the laboratory and exposed to heat, and then put in a dew chamber where infection could occur. Survival of the spores on *planta* (ability to infect and thereafter sporulate under optimal conditions) was dependent on temperature and duration of exposure. The results of the first experiment, presented in Fig 5A, show that at 15–28°C spores survived well for 24h but not for 96h. The results of the second experiment presented in Fig 5B show that the spores survived at 25°C for 48h but not at 30°C. The data obtained from the third and fourth experiments showed that 31h of exposure to 35–40°C greatly reduces survival, whereas 72h of exposure were detrimental at 30–40°C (Fig 5C).

#### Heat tolerance of splash-dispersed spores

**Fig 6:**
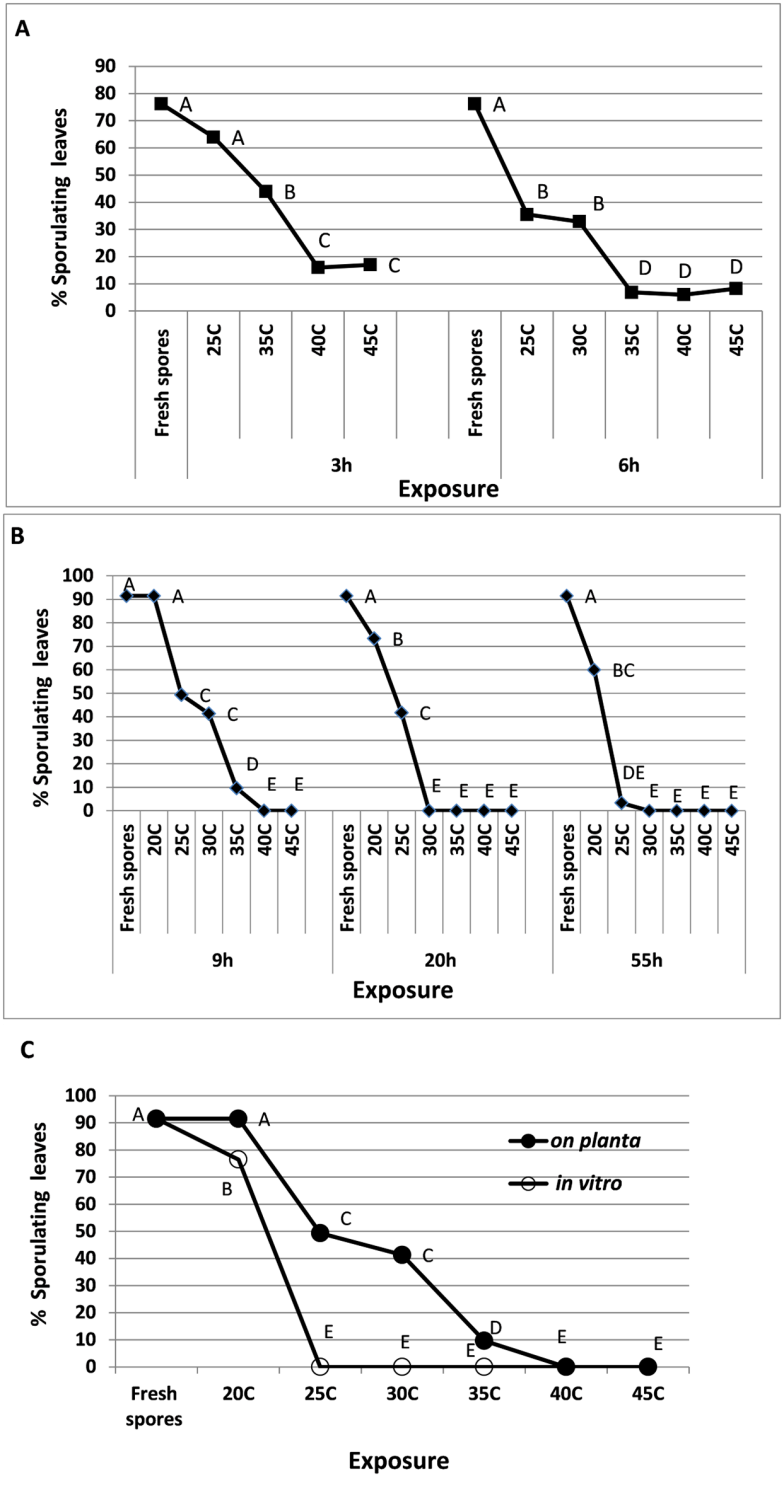
Survival of splash-dispersed spores of *P*. *belbahrii* on dry leaf surfaces of basil plants exposed to various temperatures for various periods of time. **A**- Exposure period of 3 or 6 hours. **B**- Exposure period of 9, 20 or 55 hours. **C**- Exposure period of 9 hours *on planta* or *in vitro*. Different letters on graphs indicate significant difference between means at α = 0.05 (Tukey’s HSD test).

To mimic the ability of splash-dispersed spores to survive on leaf surface in nature, spray-inoculated basil plants were exposed, immediately after inoculation, to strong air ventilation at 15–20°C to dry off the inoculum droplets (20–30 minutes) and then exposed to heat treatments at ambient humidity. As with wind-dispersed spores, survival of splash-dispersed spores *on planta* was dependent on temperature and duration of exposure (Fig 6). The results in Fig 6A show that such spores could withstand 3h of drying *on planta* at 25–45°C before being rewetted for infection, although infectious capacity declined as temperature rose. No survival was recorded after 3h at 48°C (not shown). When the drying period was extended to 6h, survival further decreased, reaching a low level at 35–45°C (Fig 6A). At longer drying periods of 9h, 20h or 55h, the spores did not survive at 40–45°C, 30–45°C, and 25–45°C, respectively (Fig 6B). Results in Fig 6C show that spores lying dry on the bottom of an open Petri dish for 9h were significantly more sensitive to heat compared to spores residing on the surface of a dry leaf. Spores that were kept dry in vitro for 4h at 30°C were capable of causing infection (with sporulation) in 11.5±5.6% of the plants inoculated, compared to 100% with fresh spores.

#### Heat tolerance of attached spores at various relative humidities

**Fig 7:**
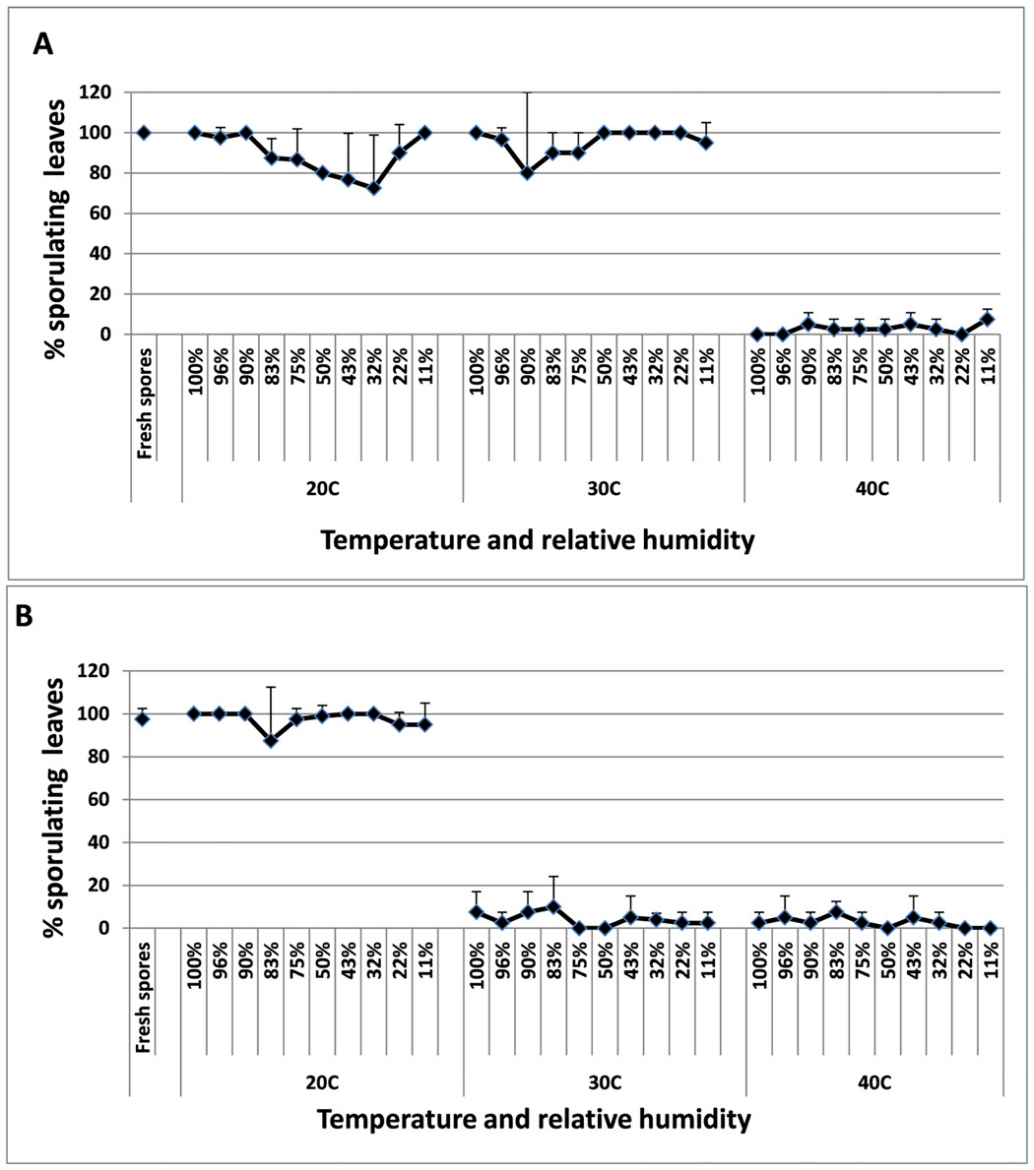
Survival of *P*. *belbahrii* spores at various RHs. Spores derived from detached inoculated leaves kept in separate containers at RHs of 11–100% and exposed to 20, 30 and 40°C, respectively. **A-** 24 hours and **B-** 48 hours. Bars indicate standard deviation of the mean.

Detached leaves, carrying freshly-produced spores of *P*. *belbahrii* were incubated at various combinations of temperature (20–40°C) and RH (11–100%) for 24h or 48h. The spores were then collected and their survival was determined by testing their ability to infect healthy basil plants. Freshly produced spores kept at 20°C at 100%RH served as positive controls. The results given in Fig 7 show that survival was not affected by RH but rather by time and temperature. At 24h (Fig 7A) spores survived variably well at 20°C and 30°C under all RH’s but very poorly at 40°C. At 48h, spores survived well at 20°C at all RH’s but very poorly at 30°C or 40°C (Fig 7B).

### Daytime solar heating: field experiments

#### Experiment A

**Fig 8:**
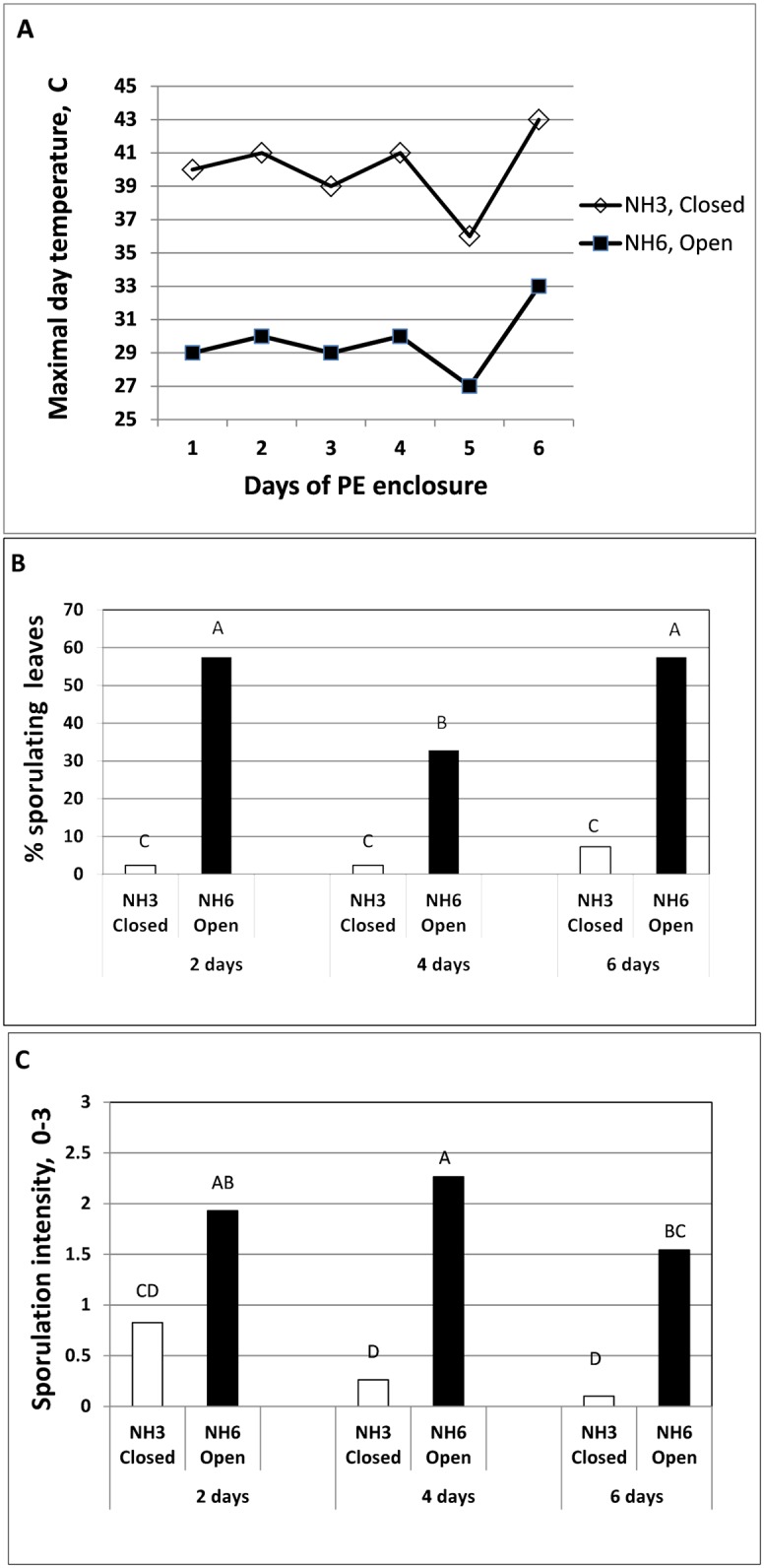
Suppression of downy mildew development by daytime solar heating applied to basil plants. **A-** Temperature data taken with HOBO data logger inserted in the mid canopy of the plants. **B**- Spore infectivity and **C**- Re-sporulation of *P*.*belbahrii*. Infected basil plants were grown in two net-houses NH3 and NH6. NH3 was fully covered with PE whereas NH6 was left uncovered at its west and east sides. Infected leaves were collected at 2, 4 and 6 days after enclosureand spores tested for infectivity and leaves for re-sporulation. Different letters on bars indicate significant difference between means at α = 0.05 (Tukey’s HSD test).

Sealing the east and west sides of net-house NH3 with PE increased the maximal daily temperature to 38–43°C as compared to 27–33°C in NH6 (Fig 8A). The spores collected from the closed house NH3 after 2, 4 or 6 days of solar heating were significantly less infectious to healthy basil plants compared to the spores collected from the open house NH6 (Fig 8B). The leaves collected from NH3 at these time intervals showed 53, 89 and 94% reduced capacity to re-sporulate as compared to leaves collected from NH6 (Fig 8C).

#### Experiment B

Enclosing the east and west sides with PE raised the maximal daily temperature in NH6 to 48–57°C as compared to 35–37°C in the adjacent, open sides NH3. Spores collected from NH6 before enclosure were highly infective with 80±10% of the inoculated leaves in a plant (n = 8) showing sporulation at 7 dpi, as compared to the spores collected at ≥3 days after enclosure which had totally lost infectivity. Similarly, the leaves sampled before enclosure showed heavy re-sporulation as against those sampled at ≥3days after enclosure which had lost their capacity to re-sporulate. Two spore trapping procedures conducted on 30.3.14 and 31.3.2014 resulted in zero infection on the 80 trap plants, suggesting that daytime solar heating had abolished the pathogen in NH6.

#### Experiment C

**Fig 9:**
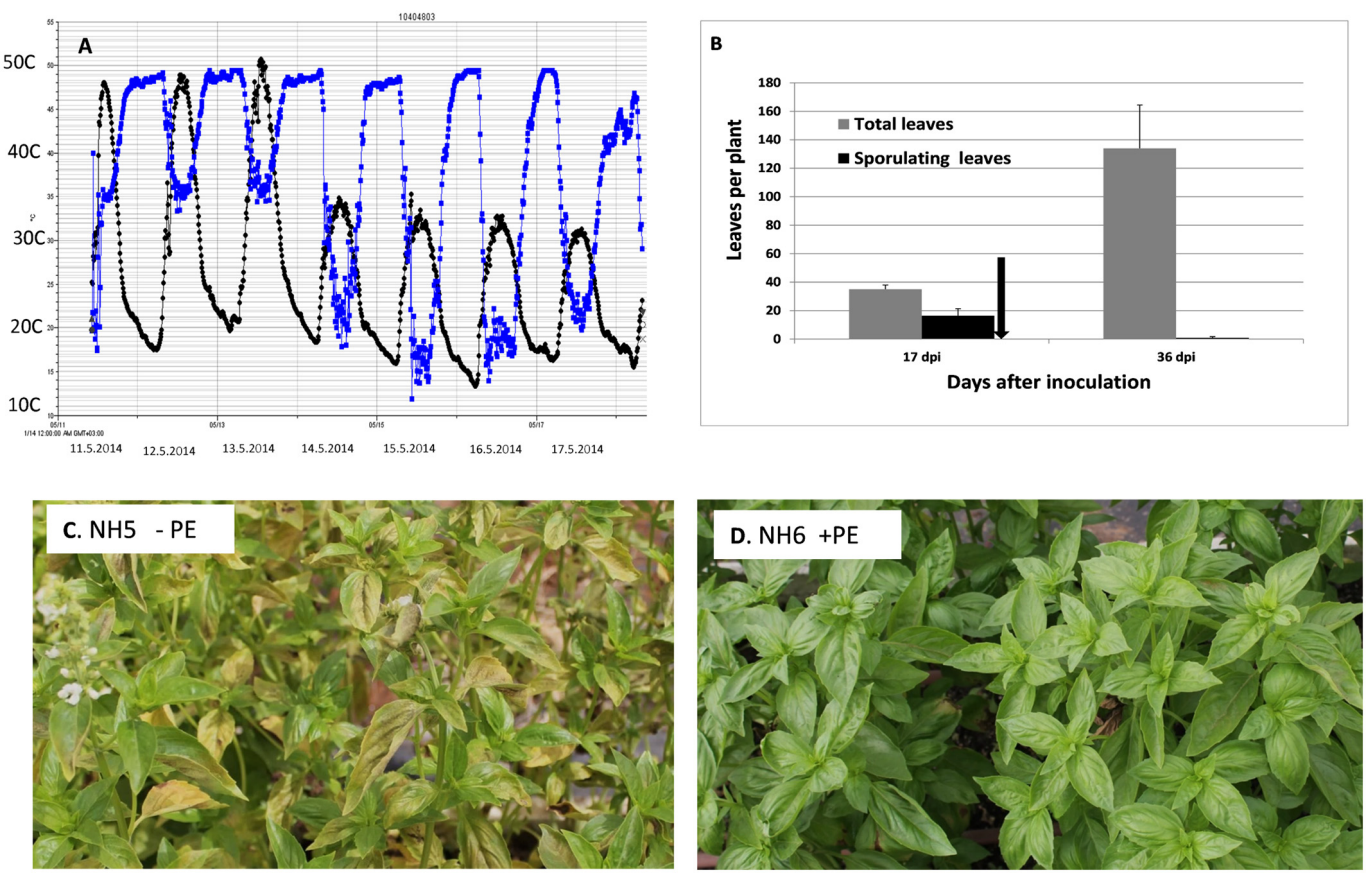
Suppression of downy mildew development by daytime solar heating applied for 3 days to inoculated basil plants. **A**- Temperature and RH data taken with HOBO data logger inserted in the mid canopy of the plants. Black line = temperature°C, blue line = %RH. **B**-Mean number of total and infected leaves per plant at 17 and 36dpi. Bars indicate standard deviation of the mean. Arrow indicates the location of solar heating on the time axis, 19-21dpi. C- Heavy disease in NH5. D- No disease in NH6. Plants in both NH5 and NH6 were inoculated with *P*.*belbahrii*. At 19 dpi NH6 was covered with PE for 3 days while NH5 remained uncovered. Photos were taken 2 weeks after removing the PE.

At 17 dpi (9.5.2014), about 83±4% of the leaves in the plants showed downy mildew symptoms. At 33 dpi, two weeks after the solar heating was terminated, percent leaves showing infection in the net-house dropped down to 1.2%. Solar heating was applied at 19 dpi (11.5.2014) for 3 days. The maximal daily temperature during PE enclosure ranged between 49–57°C; after removing the PE these figures dropped down to 30–35°C (Fig 9A black dots). The spores collected at 0, 1, 2, and 3 days after enclosure were capable of producing infection in 63±17%, 1.25±2.2%, 0%, and 0% of the leaves in the potted plants inoculated, respectively. The capacity of these sampled leaves to re-sporulate reached a mean level (0–3 visual scale) of 2.3, 0.1, 0 and 0, respectively. A few days after ending the solar heating period, enhanced desiccation of the infected leaves and appearance of new healthy leaves were apparent. Before enclosure, at 17 dpi, mean number of leaves per plant was 35, with 47% of them exhibiting symptoms of downy mildew (Fig 9B). At 33 dpi, following a 3-day enclosure period (at 19–21 dpi), mean number of leaves increased to 134 per plant with only 0.7% of them showing symptoms of downy mildew (Fig 9B). Solar heating was highly effective in suppressing disease progress and enhancing plant growth.

This is illustrated in Fig 9C and 9D. Plants in both houses NH5 and NH6 were inoculated on 22.4.2014. NH6 was covered with PE on 11.5.2014 for 3 days (19-21dpi) (see above) while NH5 was left PE-free. The photos were taken on 29.5.2014, two weeks after PE was removed from NH6. They show heavy disease in NH5 but no disease in NH6.

#### Experiment D

**Fig 10:**
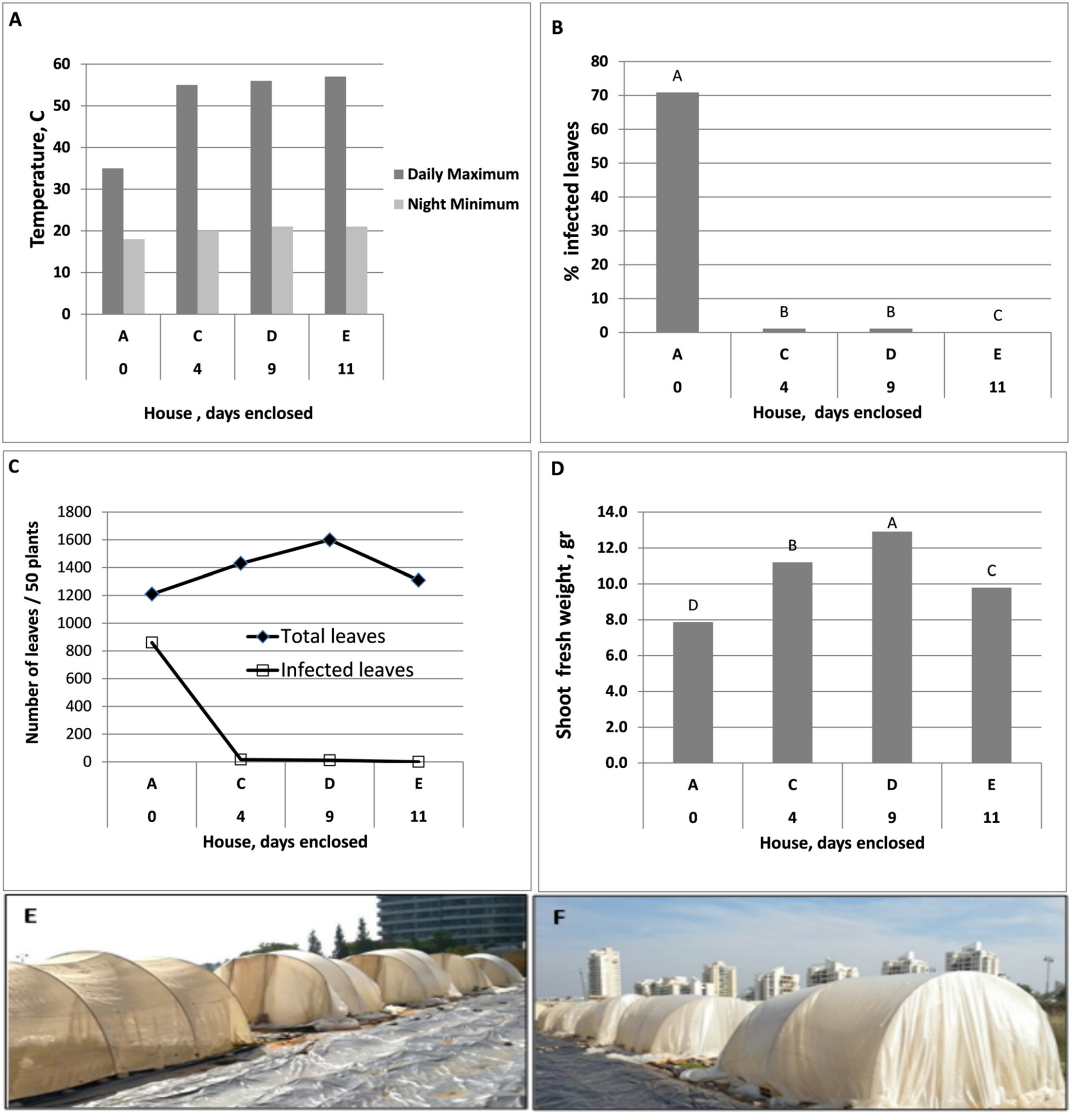
Suppression of downy mildew development by daytime solar heating applied to inoculated basil plants in three Houses C, D and E after being covered with PE for 4, 9 and 11 days, respectively compared to House A which was left uncovered. **A**- Maximal and minimal daily temperatures recorded in Houses A, C, D and E. **B**- Percentage of infected leaves per plant in Houses A, C, D, E. **C**- Number of total and infected leaves per 50 plants harvested per House 14 days after planting. **D**- Average Shoot fresh weight [g] per plant and House. Different Letters indicate significant differences between means at α = 0.05 (Tukey’s HSD test). **E**- and **F**- a general view over net houses A-E before (E) and after (F) PE coverage.

A major rise in daily maximal temperature was recorded in Houses C, D and E as a result of PE-coverage (Fig 10A). Plants grown in the control House A (PE-free) exhibited heavy sporulation of *P*. *belbahrii* with about 71% of the leaves of a plant bearing spores of the pathogen. In contrast, plants growing in Houses, C, D and E showed sporulation in 1.19, 0.75 and 0.08% of the leaves, respectively (Fig 10B). The high temperature imposed by PE coverage enhanced plant growth in Houses C, D and E compared to House A (Fig 10C), and significantly increased shoot fresh weight (Fig 10D). The pictures in Fig 10E and 10F show houses before and after enclosure, respectively.

#### Experiment E

**Fig 11:**
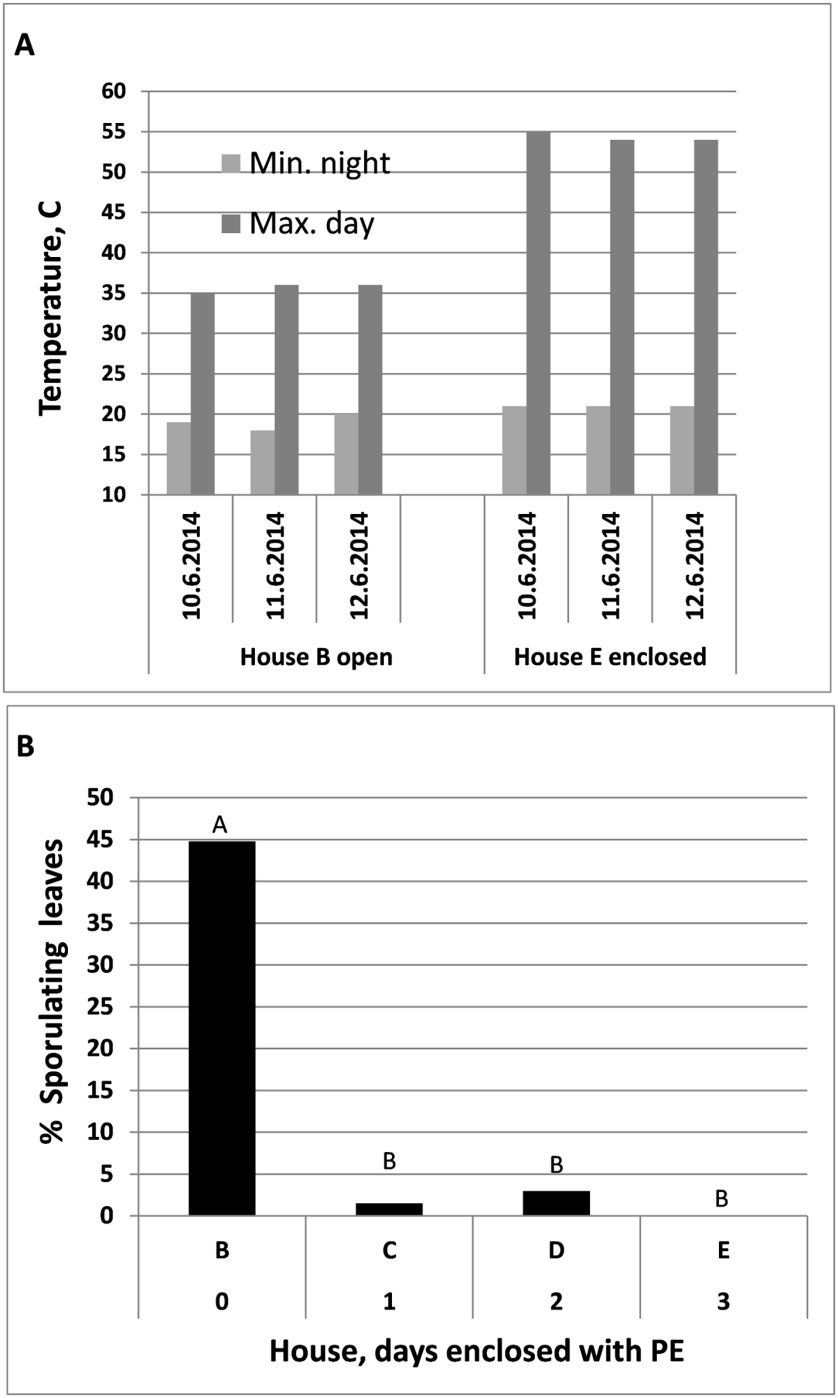
Suppression of downy mildew development by daytime solar heating applied to inoculated basil plants in three Houses C, D and E after being covered with PE for 1, 2 and 3 days, respectively, immediately after inoculated plants have been transplanted in the Houses. House B was PE-uncovered and served as control. **A**- Minimal and Maximal Temperatures in Houses E and B (control). **B**- Percentage of sporulating leaves of plants uprooted from Houses B, C, D and E at 8 dpi and incubated over night for sporulation assessment. Different letters indicate significant differences between means at α = 0.05 (Tukey’s HSD test).

Maximal daily temperatures in the PE-enclosed House E were 55°C, 54°C, and 54°C at 1, 2 and 3 days of enclosure, respectively, as compared to 35°C, 36°C and 36°C in the control uncovered House B (Fig 11A). Maximal day temperature dropped down to 36°C at 1 and 2 days after removing the PE cover. Disease development was significantly suppressed in all three PE-covered houses (C, D and E) as compared to the control PE-uncovered House B (Fig 11B). Percent leaves showing sporulation of *P*. *belbahrii* in the houses enclosed for 0, 1, 2 and 3 days was 44.8, 1.5, 3.0 and 0%, respectively (Fig 11B).

#### Experiment F

**Fig 12:**
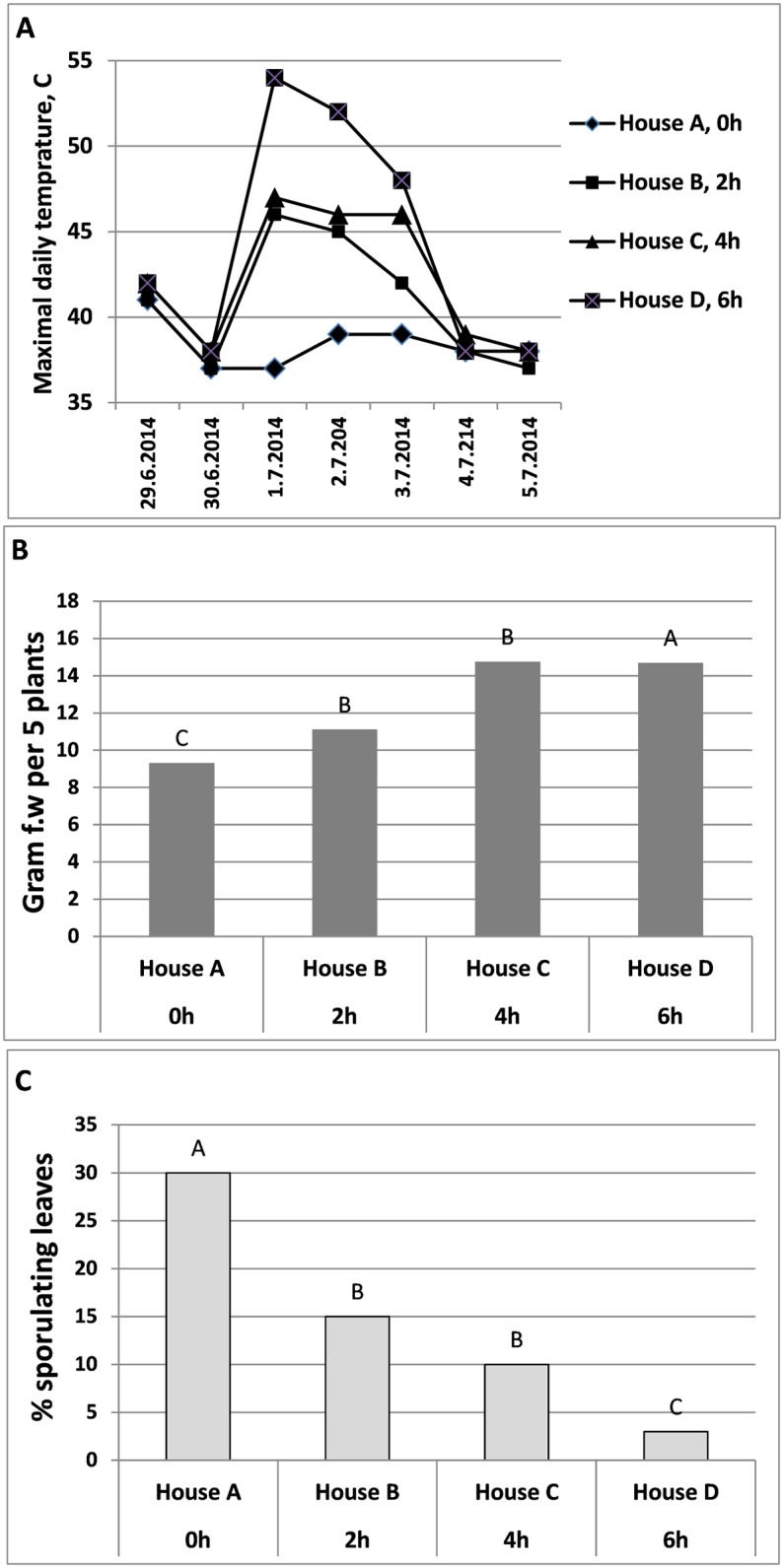
Suppression of downy mildew development by daytime solar heating applied to inoculated basil plants in three Houses B, C and D being covered with PE for 2, 4, or 6 hours, respectively at three consecutive days. House A was PE-uncovered and served as control. **A**- Maximal daily temperature during the experiment in Houses A, B, C and D. **B**- Mean shoot fresh weight [g] per 5 plants grown in Houses A, B, C and D at 24 dpi. **C**- Percentage of sporulating leaves per plant uprooted from Houses A, B, C and D at 23 dpi and incubated over night for sporulation assessment. Different letters indicate significant differences between means at α = 0.05 (Tukey’s HSD test).

High temperatures (max. 41–42°C) prevailed on the day of planting. PE coverage for 3 days raised the daily maximal temperature. It was 37–39°C in the control House A, compared to 42–46°C, 46–47°C and 48–54°C, in Houses B, C, and D, respectively (Fig 12A). Mean shoot fresh weight was significantly higher in Houses B, C, and D compared to the control House A (Fig 12B). Disease development was significantly reduced as a result of PE-coverage, more so in House D (PE for 6h/day) compared to House B (PE for 4h/day) or House C (PE for 2h/day) (Fig 12C). The control plants that remained indoors at 25°C were fully blighted from the disease.

#### Experiment G

**Fig 13:**
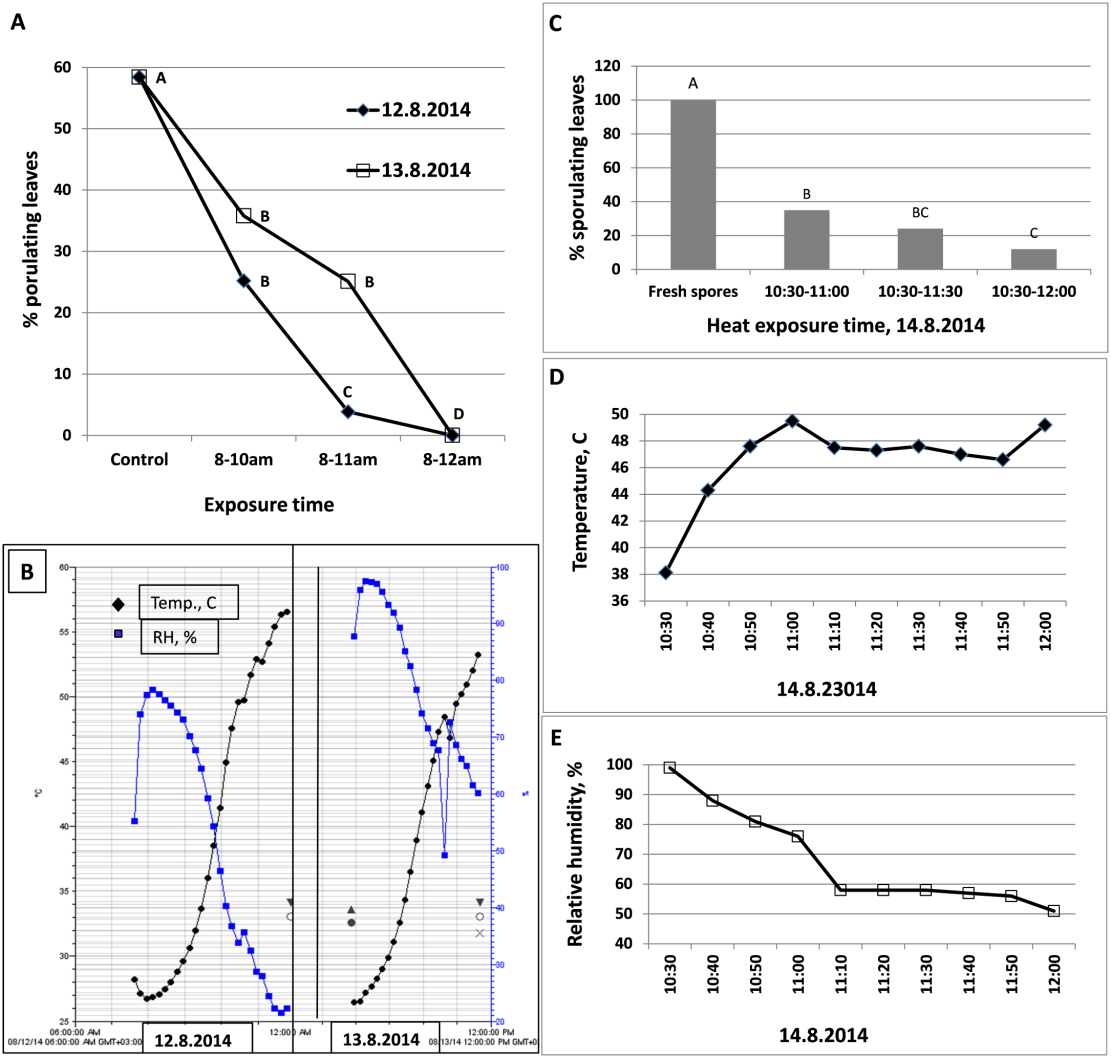
Suppression of downy mildew development by daytime solar heating applied to inoculated basil plants in House F. **A**- Sporulation of the pathogen on plants after exposure to heat for 2, 3, or 4 h at 6dpi (12.8.2014) or at 7dpi (13.8.2014). **B**- Air temperature and relative humidity in House F during the experimental period on 12.8.2014 and 13.8.2014. **C**- Infectivity of attached spores after exposure to heat for 30, 60 or 90 minutes in House F on 14.8.2014. **D**- Air temperature, and **E**- Relative humidity, in House F during the experimental period on 14.8.2014.

Survival of the mycelium gradually declined with time, more so on 12.8.2014 than on 13.8.2014. In both days, exposure to solar heating from 8 am until noon was detrimental to the pathogen (Fig 13A). On 12.8.2014, air temperature rose from 28°C at 8 am to 56.5°C at 12 noon. RH declined from 60% at 8 am to 20% at 12 noon (Fig 13B). On 13.8.2014, air temperature rose from 24°C to 53.5°C and RH declined from 98% to 60% (Fig 13B). Survival of attached spores exposed to solar heating on 14.8.2014 is shown in Fig 13C. Exposure of 30, 60 or 90 minutes to heat and reduced RH (Fig 13D and 13E) reduced spore survival (infectivity) significantly, by 65, 76 and 88%, respectively (Fig 13C).

#### Experiment H

**Fig 14:**
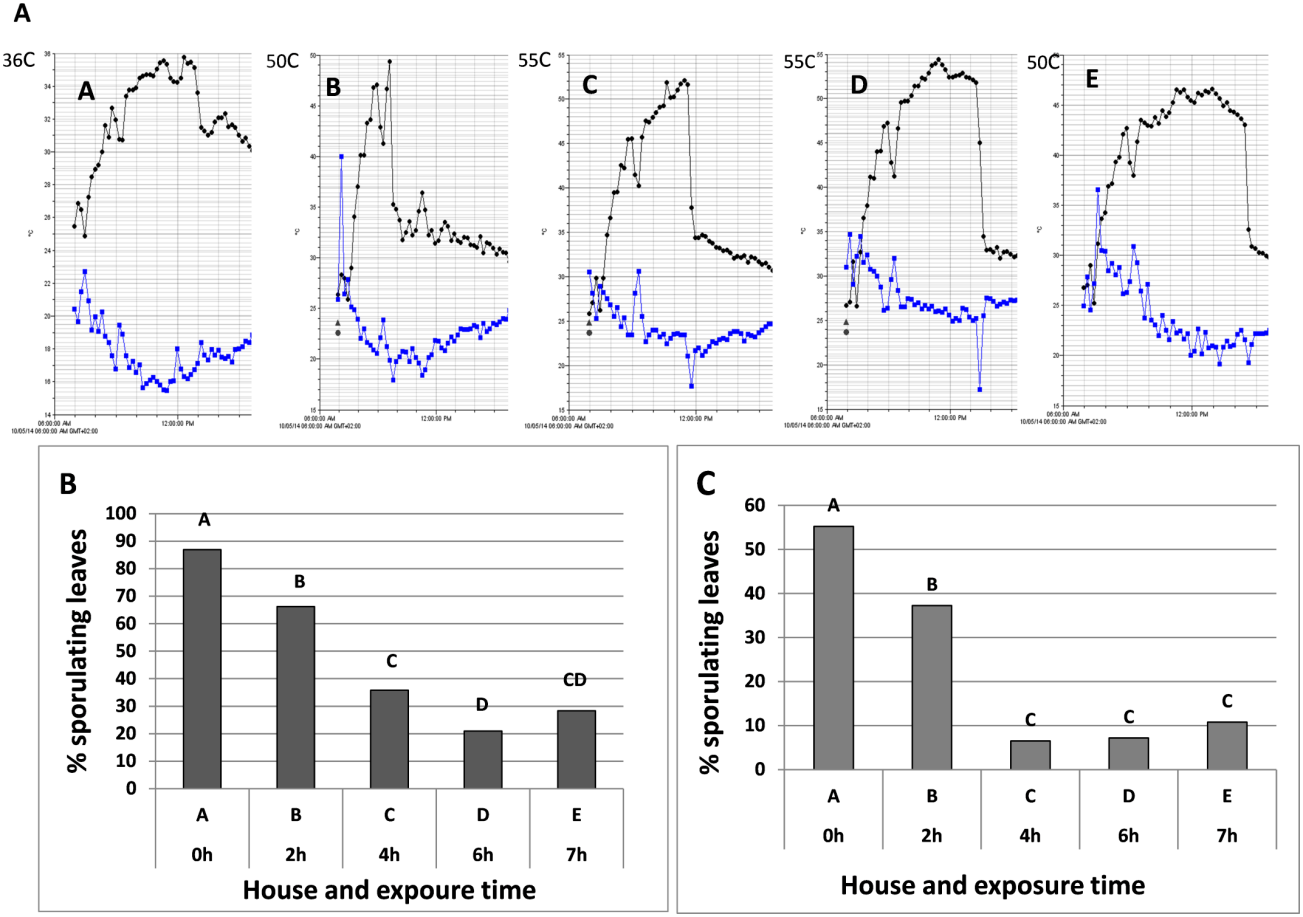
Suppression of downy mildew development by daytime solar heating applied to infected basil plants (6 dpi) in four Houses B, C, D and E being covered with PE for 2, 4, 6 or 7 hours, respectively during a single day. House A was PE-uncovered and served as control. **A**- Temperature (black lines) and RH (blue lines) during the experiments in Houses A-E (please note the different temperature scales). **B**- Percentage of sporulating leaves two days after exposure of plants to heat (8 dpi) in Houses A-E. Different letters indicate significant differences between means at α = 0.05 (Tukey’s HSD test). **C**- Percent sporulating leaves of potted basil plants placed as traps for 24 hours in Houses A-E and thereafter being incubated for 20 h in a dew chamber to promote infection. Sporulation was assessed 7 dpi. Different letters indicate significant differences between means at α = 0.05 (Tukey’s HSD test).

Maximal daily temperature in the control House A was 35.5°C, whereas during the solar heating period maximal daily temperature in Houses B, C, D, and E rose to 49.5, 52, 54.5 and 46.5°C, respectively (Fig 14A black dots). Ability of the pathogen to sporulate on infected plants declined significantly as a result of solar heating (Fig 14B). Percent leaves per plant showing sporulation in the control House A was 87% while it was 24, 59, 76 and 68% lower in Houses B, C, D and E, respectively. Infection of the trap plants (Fig 14C) confirmed that sporulation and/or spore viability were significantly suppressed as a result of solar heating.

## Discussion

Control of downy mildew in basil may be achieved not only by chemical means but also by physical means. Nocturnal illumination was recently shown to be one effective control measure for this disease. Illumination during the night inhibits spore formation of the causal agent *P*. *belbahrii* and thus suppresses its epidemic progress [4]. Ventilation of basil crops growing in a greenhouse or a plastic houses is another effective measure. *P*. *belbahrii*, like other downy mildew pathogens, requires free leaf moisture for infection and high ambient relative humidity for sporulation. Ventilation reduces air humidity and avoids vapor deposition on leaf surface, thus preventing infection and sporulation.

In this paper we explored the feasibility of another physical measure, daytime solar heating, to control the disease.

The tolerance/sensitivity of downy mildews pathogens to heat and relative humidity (RH) has been extensively studied primarily for sporangia. Survival of these pathogens generally is affected by high temperature (> 35°C) and low humidity. An exception is *Peronospora tabacina*, the causal agent of tobacco downy mildew. Sukanya and Spring [6] showed that sporangia attached to sporulating leaf tissue of tobacco, survived 48h at 50°C; detached sporangia (vacuum sucked) survived 24h at 70°C. In another study, survival of *P*. *tabacina* inside tobacco leaves was lost after exposure for 24h to 35–45°C or 72h to 30°C [7]. Bashi and Aylor [8] studied the effect of temperature and RH on survival of detached sporangia of *Peronospora destructor*, the causal agent of onion downy mildew. Sporangia were exposed for various lengths of time to different laboratory combinations of temperature and RH; temperatures of 10, 25, and 35°C were used in combination with 33, 53, 76, and 95% RH. Germination indicated survival. For all RH tested, *P*. *destructor* sporangia survived best at 10°C and poorest at 35°C. For all temperatures tested, sporangia survived poorest at 33% RH, and differences in survival between 53 and 76% RH were not significant.

Sporangia of *Bremia lactucae*, the causal agent of lettuce downy mildew, survived much longer at 23°C (>12 h) than at 31°C (2 to 5 h), regardless of RH (33 to 76%) [9].

Kennelly et al. [10] studied survival of sporangia of *Plasmopara viticola*, the grapevine downy mildew pathogen, under field condition. In fair weather, most sporangia died sometime during the daylight period immediately following their production. However, over 50% of sporangia still released zoospores after 12 to 24h of exposure to overcast conditions.

Exposure of potato leaves infected with the late blight pathogen *Phytophthora infestans* for 12h to 40°C or 36h to 35°C were lethal to the mycelium inside the leaves [11]. Air-dispersed sporangia of *P*. *infestans* survived on leaves of intact potato plants up to 8h at 30°C whereas attached sporangia survived up to 24h under the same conditions [11]. Studies done with *Pseudoperonospora cubensis*, the agent of downy mildew in cucumber, showed [12] that air-dispersed sporangia lying on intact cucumber leaves could survive 22h at 35°C but not at 40°C.

The present study provides data on the sensitivity of *P*. *belbahrii* to heat; the data collected from field trials documented that daytime solar heating may be a feasible method to control the downy mildew disease it causes in sweet basil.

While the maximal temperature allowing infection, colonization and sporulation of *P*. *belbahrii* are known (27°C, 31°C, and 29°C, respectively, Y. Cohen, unpublished data), the maximal temperature for survival of its mycelium inside mildewed basil leaves or its spores (attached or dispersed) had not been studied. Here we show that exposure of infected basil plants at 7 dpi to heat (30–45°C) may be detrimental to sporulation of *P*. *belbahrii* on such plants. While a single 3h exposure to heat had a minor effect on survival of mycelium, three 3h consecutive daily exposures, or one longer exposure were strongly suppressive, especially at high temperatures. Thus, mycelium inside leaves did not survive exposure of 6-9h at 40–45°C, 17h at 35–45°C, or 27h at 30–45°C. Exposure to heat was less suppressive when applied to infected plants at 5 dpi compared to plants at 7 dpi or 9 dpi. This may be attributed to either reduced sensitivity to heat of young mycelium, the two days recovery period that 5 dpi plants experienced in the experiment, or both. However, similar recovery period provided to 7 or 9 dpi plants did not restore sporulation.

Sensitivity to heat of spores of *P*. *belbahrii* was determined by testing their ability to infect, and subsequently sporulate, on healthy plants under optimal conditions. Spores attached to infected plants lost infectivity after exposure of 12h to 35–45°C. Spores attached to whole plants and kept in moistened plastic boxes or at ambient growth-chamber humidity retained some infectivity after exposure of 22h to 35–45°C, but lost it after 72h.

Naturally-dispersed spores lying on healthy, dry basil plants retained some infectivity after exposure to 35–40°C for 31h, but lost it after exposure to 30–40°C for 72h. Infectivity after exposure to 25°C for 48h was low (about 20% sporulating leaves) and infectivity after exposure to 30°C for 48h was lost.

To test the wetting-drying effect on spore survival, spray-inoculated plants were quickly dried-off by air ventilation (within 20–30 minutes at 20°C) and exposed to heat treatments. Unlike *P*. *cubensis* or *P*. *infestans* whose sporangia fail to survive after a wetting-drying process [12], wetted and dried spores of *P*.*belbahrii* survived on the surface of basil leaf for up to 55h, depending on temperature. They lost infectivity after 9h at 40–45°C, 20h at 30–45°C, or 55h at 25–45°C. Survivability of such spores was higher *on planta* than *in vitro*.

The laboratory-generated data suggested that spores and mycelium of *P*. *belbahrii* are quite sensitive to heat. Therefore, we used this finding to control *P*. *belbahrii* in basil plants growing outdoors. An effective way to warm up the environment inside a growth house is to utilize the ‘greenhouse effect‘, namely, capture the solar energy by covering the house with a transparent, infra-red-impermeable polyethylene (PE) sheet. We show here that PE coverage raised the daily maximal temperature by 11–22°C relative to non-covered house, depending on the experiment/season. In six field experiments conducted on Nov.20, 2013, May 9, 2014, May 11, 2014, June 7, 2014, June 29, 2014 and Sept. 29.2014, the temperature within the basil canopy in PE-covered houses was elevated on average by 11, 16, 22, 20, 17, and 20°C, respectively, compared to uncovered houses. This rise in temperature resulted, in all experiments, in a significant decline in survival of the pathogen, suppression or complete halt of disease development, and in some experiments, with a re-habilitation of the crop. Daytime solar heating not only suppressed downy mildew progress but also enhanced plants growth.

Our results corroborate with Elad et al [13] who used daytime solar heat treatments to suppress powdery mildew in pepper and tomato and gray mold in sweet basil. Temperatures in polyethylene-covered sweet basil greenhouses that were kept closed for 6 h each day reached 42°C during the winter period. The incidence of gray mold (*Botrytis cinerea*) in these greenhouses was significantly reduced and basil yields increased. A significant negative correlation was observed between the duration of temperatures above 30°C and the incidence of gray mold [13].

Our results also agree with Bitton and Silverman [14] who recommended using daytime solar heating for the control of *Thrips tabaci* in chives herb plants (*Allium schoenoprasum*) in greenhouses in Israel. The plants are covered with PE until temperature reaches 45–46°C, or ≥40°C for 2-3h. This treatment may be repeated in intervals of 3–5 days [14].

In conclusion, daytime solar heating may be used to control downy mildew in basil crops growing in greenhouses or net-houses. Solar energy may be captured by shutting/closing down glass windows, or covering the house with a transparent IR polyethylene sheet during sunny hours of the day. This should be done cautiously to avoid heat damage, especially to young plants. We suggest that crops should be inspected routinely and the practice implemented at first sign of the disease. Best is to use 3 consecutive daily exposures of 3–4 hours starting at 8 am. Growers should monitor temperature and uncover early if temperature becomes too hot (>45°C) or add another treatment day if temperature does not become sufficiently high (<35°C). Solar heating could be used as a routine practice implemented for crops with a low tolerance for the disease.

In organic farming, daytime solar heating combined with nocturnal illumination [4] could control downy mildew adequately without fungicide applications. In conventional farming, integration of daytime solar heating in disease management program may reduce the number of fungicide applications needed.
